# Research on deep reinforcement learning basketball robot shooting skills improvement based on end to end architecture and multi-modal perception

**DOI:** 10.3389/fnbot.2023.1274543

**Published:** 2023-10-13

**Authors:** Jun Zhang, Dayong Tao

**Affiliations:** ^1^Department of Physical Education and Research, Lanzhou University of Technology, Lanzhou, China; ^2^Department of Physical Education, Guilin Normal College, Guilin, China

**Keywords:** end-to-end architecture, multi-modal perception, deep reinforcement learning, basketball robot, improved shooting skills

## Abstract

**Introduction:**

In the realm of basketball, refining shooting skills and decision-making levels using intelligent agents has garnered significant interest. This study addresses the challenge by introducing an innovative framework that combines multi-modal perception and deep reinforcement learning. The goal is to create basketball robots capable of executing precise shots and informed choices by effectively integrating sensory inputs and learned strategies.

**Methods:**

The proposed approach consists of three main components: multi-modal perception, deep reinforcement learning, and end-to-end architecture. Multi-modal perception leverages the multi-head attention mechanism (MATT) to merge visual, motion, and distance cues for a holistic perception of the basketball scenario. The deep reinforcement learning framework utilizes the Deep Q-Network (DQN) algorithm, enabling the robots to learn optimal shooting strategies over iterative interactions with the environment. The end-to-end architecture connects these components, allowing seamless integration of perception and decision-making processes.

**Results:**

The experiments conducted demonstrate the effectiveness of the proposed approach. Basketball robots equipped with multi-modal perception and deep reinforcement learning exhibit improved shooting accuracy and enhanced decision-making abilities. The multi-head attention mechanism enhances the robots' perception of complex scenes, leading to more accurate shooting decisions. The application of the DQN algorithm results in gradual skill improvement and strategic optimization through interaction with the environment.

**Discussion:**

The integration of multi-modal perception and deep reinforcement learning within an end-to-end architecture presents a promising avenue for advancing basketball robot training and performance. The ability to fuse diverse sensory inputs and learned strategies empowers robots to make informed decisions and execute accurate shots. The research not only contributes to the field of robotics but also has potential implications for human basketball training and coaching methodologies.

## 1. Introduction

Basketball is one of the most popular and influential sports globally, characterized by its competitive, recreational, and fitness aspects, as well as its rich cultural and social values (William and Alex, 2023). With the continuous advancement of technology and evolving societal needs, basketball faces new challenges and opportunities (Li and Xu, 2021). How can artificial intelligence be leveraged to enhance the skills and tactics of basketball players and coaches? How can AI contribute to enriching the viewing experience and engagement of basketball audiences and learners? How can AI expand the application and impact of basketball in areas such as entertainment, education, and healthcare? These questions have sparked our interest and enthusiasm for researching basketball robots.

A basketball robot is an intelligent agent capable of emulating human shooting behaviors in real-world scenarios. It can autonomously adjust shooting angles, force, direction, and timing according to different environmental conditions and task requirements, achieving high-precision shooting goals (Zhi and Jiang, 2020). Basketball robots possess several characteristics: (1) They are multi-degree-of-freedom, nonlinear, and underconstrained mechanical systems, influenced by various factors such as gravity, air resistance, and friction. (2) They are multi-modal, multi-scale, and multi-dimensional perception systems, integrating visual, motion, distance, and other modalities, spanning near-field, far-field, and full-court perception. (3) They are complex, uncertain, and dynamic decision-making systems, requiring comprehensive considerations of factors such as shooting targets, strategies, and outcomes, while adapting to real-time changes in the environment and tasks.

Basketball robots, as typical representatives of intelligent agents, hold promising application prospects and significant research value. They can serve as auxiliary tools for coaches and players in basketball matches, providing real-time tactical analysis and technical guidance. Additionally, they can be utilized in entertainment and education, showcasing high-level basketball skills to audiences and students. With the continuous advancement of technology, basketball robots are poised to become important subjects of research in the field of intelligent agents, further expanding their application scope in real-life scenarios.

However, enhancing the shooting skills of basketball robots in real-world settings still faces multiple challenges (Siegel and Morris, 2020). Traditional methods often require manual design and processing of features, rules, and parameters, neglecting the interaction between perception and decision-making processes, which limits the application of basketball robots in complex environments. Therefore, this study aims to introduce an end-to-end architecture, unifying the perception and decision-making processes into a single learning system, to achieve efficient shooting behavior in basketball robots.

Over the past few years, with the rise of deep learning, the field of basketball robots has achieved several significant research breakthroughs. Existing studies have mostly focused on the application of single technical approaches, such as traditional control algorithms and visual tracking techniques. While these methods have demonstrated certain effectiveness in specific scenarios, they still have limitations in dealing with complex environments and integrating multi-modal information (Yao et al., 2023). In recent years, end-to-end learning has become a popular direction in artificial intelligence, achieving higher-level task resolution by learning the mapping from raw inputs to final outputs (Zhao et al., 2022). However, the application of end-to-end architecture in enhancing the shooting skills of basketball robots has not been widely explored. Multi-modal perception, as a method of integrating various sensory information (Wu et al., 2022), has also gained increasing attention in the field of robotics. By simultaneously fusing visual, motion, and distance information (Hong et al., 2020), multi-modal perception enables robots to have a comprehensive understanding of the environment and tasks, thus improving decision-making accuracy (Ince et al., 2021). Nevertheless, research on multi-modal perception in enhancing the shooting skills of basketball robots is still relatively limited. In the domain of deep reinforcement learning, the DQN algorithm, as a classic reinforcement learning method, has achieved remarkable success in various fields (Hong et al., 2021). However, its application to enhance the shooting skills of basketball robots requires addressing challenges such as high-dimensional state space and sparse reward signals, to achieve efficient learning and optimization of robots in complex environments.

In the literature review and survey of relevant fields, it was found that existing research on basketball robots mainly focused on motion control and path planning. The application of end-to-end learning and multi-modal perception in improving shooting skills was relatively limited. Le et al. (2022) proposed the CodeRL to address the limitations of end-to-end learning methods in ignoring validation information. However, the introduction of end-to-end architecture enables robots to efficiently learn shooting strategies, and the adoption of multi-head attention mechanism enhances the robot's perception capabilities in complex environments, providing comprehensive information for shooting decisions. Deep learning can complete decision-making tasks, like Counterfactual examples (CFs) (Chen et al., 2022), and human interaction (Akalin and Loutfi, 2021). A multi-agent deep learning is proposed to implementation of robust equilibrium (RE) (He et al., 2023).

Based on the aforementioned literature review and the identified research gaps, this study aims to propose an end-to-end architecture that unifies the perception and decision-making processes, along with the application of multi-modal perception and the DQN algorithm to enhance the shooting skills of basketball robots. This comprehensive approach is expected to address some of the shortcomings in existing research and explore new application prospects in the field of basketball robots. Throughout the research process, we will validate the effectiveness of the proposed methods through extensive simulation experiments and real-world tests. Through the analysis and summarization of the experimental results, we hope to gain new insights and inspirations in the enhancement of basketball robot shooting skills, providing fresh ideas and directions for the development of intelligent agent control.

In conclusion, the objective of this paper is to enhance the shooting skills of basketball robots by introducing an end-to-end architecture, multi-modal perception, and the DQN algorithm. As a paradigm of applying artificial intelligence technology in sports, basketball robots not only contribute to improving their own skill levels but also open up new possibilities for the application and development of intelligent agents in the real world. Through this research, we aspire to make a contribution to the advancement of basketball robots and provide new ideas and directions for the application of artificial intelligence technology in sports and other domains.

The contributions of this paper can be summarized in the following three aspects:

1. We apply the end-to-end architecture to improve the shooting skills of basketball robots. Traditional methods usually split the perception and decision-making processes into multiple independent modules, requiring manual design and optimization, resulting in complex systems with low efficiency. By introducing an end-to-end architecture, we fuse the perception and decision-making processes into a unified learning system, enabling the basketball robot to learn the shooting decision-making strategy more efficiently from raw input data, thereby significantly reducing the complexity of system design and optimization.
2. We use multi-head attention to process vision, motion and distance inputs for a basketball robot from different sources. It assigns weights across modalities, addressing shortcomings of traditional methods. Multi-head attention enables a more comprehensive and fine-grained understanding of environment and ball state for the robot. It helps distinguish modalities and extract features more accurately. Compared to traditional methods, multi-head attention processing of multi-modal information enables more efficient and accurate shooting decisions by the basketball robot, demonstrating benefits for perception and task performance.
3. We apply deep reinforcement learning (DRL) to enhance the shooting skills of a basketball robot. Utilizing the DQN (Deep Q-Network) algorithm, the robot learns and refines its shooting strategy through interactions with the environment, leading to gradual improvements in shooting proficiency. The DQN algorithm effectively handles this complexity using a deep neural network structure. This empowers basketball robots to efficiently learn and master intricate shooting techniques.

The logical structure of this paper is as follows: In the second section, the current research on the improvement of shooting skills of basketball robots is reviewed from three aspects: traditional methods, end-to-end learning methods and multi-modal perception methods, and the advantages and disadvantages of various methods are analyzed. An end-to-end architecture will be adopted, combined with methods of multi-modal perception and deep reinforcement learning to improve shooting skills. In the third section, the key methods used in this research, such as end-to-end architecture, multi-head attention mechanism, and DQN algorithm, are introduced in detail, and the calculation formula and flow chart of each method are given, and the design ideas, principles, and target. In the fourth section, describe the experimental environment, including hardware configuration and software environment; introduce the data set used in the experiment; define the evaluation indicators used in the experiment; outline the ideas of comparative analysis of the experiment. In the fifth section, the significance, contribution and limitations of the experimental results are summarized and discussed; the implications of the results for related research fields are discussed; the future research directions and application prospects are prospected. In the sixth section, summarize the research content, innovations and contributions of the full text; emphasize the significance of the research and its impact on related fields; look forward to the future development direction and application prospects.

## 2. Related work

Basketball has always been a sport filled with charm, and the emergence of basketball robots has brought new possibilities to this field. With the advancement of modern artificial intelligence technology, we can continuously improve the shooting skills of basketball robots through methods like reinforcement learning. This study aims to explore an innovative approach by combining end-to-end architecture, multi-modal perception, and deep reinforcement learning, enabling basketball robots to achieve more efficient and precise shooting actions in real-world scenarios. In the past, enhancing the shooting skills of basketball robots has been a challenging task, as traditional methods often required complex manual design and processing, limiting their applications in complex environments (Shi et al., 2019). Therefore, this research introduces the concepts of end-to-end architecture and multi-modal perception, combined with deep reinforcement learning algorithms, to achieve breakthroughs in enhancing the shooting skills of basketball robots.

In the basketball domain, the application of artificial intelligence has gradually become a research hotspot. As mentioned in Li and Zhang (2021), artificial intelligence technology plays a significant role in the analysis of basketball team and player performance, match result prediction, shooting analysis, and intelligent coaching systems. On the other hand, Zhu and Chen (2022) investigated the application of data mining algorithms in basketball robot target recognition, contributing to the quick and accurate target localization of basketball robots. This study focuses on enhancing the shooting skills of robots and has been explored in some related research, which can be classified based on technological approaches or research directions. This paper will review and analyze the following three aspects: (1) traditional methods, (2) end-to-end learning methods, and (3) multi-modal perception methods.

Traditional methods refer to those approaches that use traditional control algorithms and computer vision techniques to improve the shooting skills of basketball robots. These methods require manual feature extraction and complex control strategies. For example, Wei (2021), a combination of binocular stereo vision and monocular vision was used to achieve rapid recognition and localization of targets such as the court, basketball hoop, and ball. Parameters of the model were optimized using a genetic algorithm, and a hybrid control strategy combining PID control and fuzzy control was designed for shooting. This approach demonstrated certain improvements in the robot's perception ability in complex scenes and shooting accuracy. However, it also has some drawbacks, such as poor adaptability to environmental changes, weak capability of multi-modal information fusion, and limited optimization ability for shooting strategies. In various research studies, the integration of Graph Neural Networks (GNNs) with reinforcement learning has been proposed as a powerful approach for tasks such as graph interpretation (Le et al., 2022), instance prediction (Shan et al., 2021), and defense against node injection poisoning attacks (NIPA) (Sun et al., 2020) across multiple domains.

The end-to-end learning methods refer to those approaches that use deep neural networks to enhance the shooting skills of basketball robots. These methods achieve end-to-end learning from perception to decision by learning the mapping relationship from raw inputs to final outputs. For instance, in Singh et al. (2019), an end-to-end learning approach was proposed, where raw images were taken as inputs, and discrete actions were directly outputted, achieving end-to-end learning from perception to decision. This method also utilized a multi-head attention mechanism to integrate various perceptual information, such as visual, motion, and distance, thereby enhancing the robot's understanding of the environment and tasks. Furthermore, appropriate reward functions and experience replay mechanisms were designed to optimize the robot's shooting strategies, improving learning efficiency and shooting success rate. This approach demonstrated significant improvements in learning efficiency and shooting success rate of the robot. However, there are still some challenges, such as dealing with high-dimensional state space and sparse reward signals, and selecting appropriate network structures and parameters.

The multi-modal perception methods refer to those approaches that enhance the shooting skills of basketball robots by simultaneously fusing multiple perceptual information. These methods enable the robot to have a more comprehensive understanding of the environment and tasks, thus improving decision accuracy. For example, in Gu et al. (2016), a multi-modal perception approach was employed, which simultaneously fused visual, motion, and distance information, enhancing the robot's understanding of the environment and tasks. This method used a multi-head attention mechanism to weight different modalities of information, achieving dynamic information selection and integration. It also demonstrated good results in improving the robot's perception ability in complex scenes and decision accuracy for shooting. However, there are some challenges in handling multi-modal information representation and fusion, as well as designing and optimizing the multi-head attention mechanism.

In conclusion, this paper aims to explore a novel approach to enhance the shooting skills of basketball robots by integrating end-to-end architecture, multi-modal perception, and deep reinforcement learning methods. Compared to previous research, this study combines the perception and decision-making processes into a unified learning system, enabling the robot to perform shooting actions more efficiently and accurately in complex environments. Furthermore, the introduction of multi-modal perception utilizes the multi-head attention mechanism to fuse visual, motion, and distance cues, enhancing the robot's perception capabilities in complex scenes and improving shooting decision accuracy. The most significant innovation lies in the adoption of the deep reinforcement learning's DQN algorithm, allowing the basketball robot to learn superior strategies through interactions with the environment, progressively improving shooting skills, and optimizing shooting performance. The introduction of the DQN algorithm enables the robot to learn and master complex shooting actions more efficiently.

By integrating end-to-end architecture, multi-modal perception, and deep reinforcement learning, this study provides a new perspective and method to enhance the shooting skills of basketball robots. The achievements of this research are believed to make significant contributions to the development of intelligent agent control and offer valuable insights for the future application of deep learning in robot tasks.

## 3. Methodology

**Figure 1:**
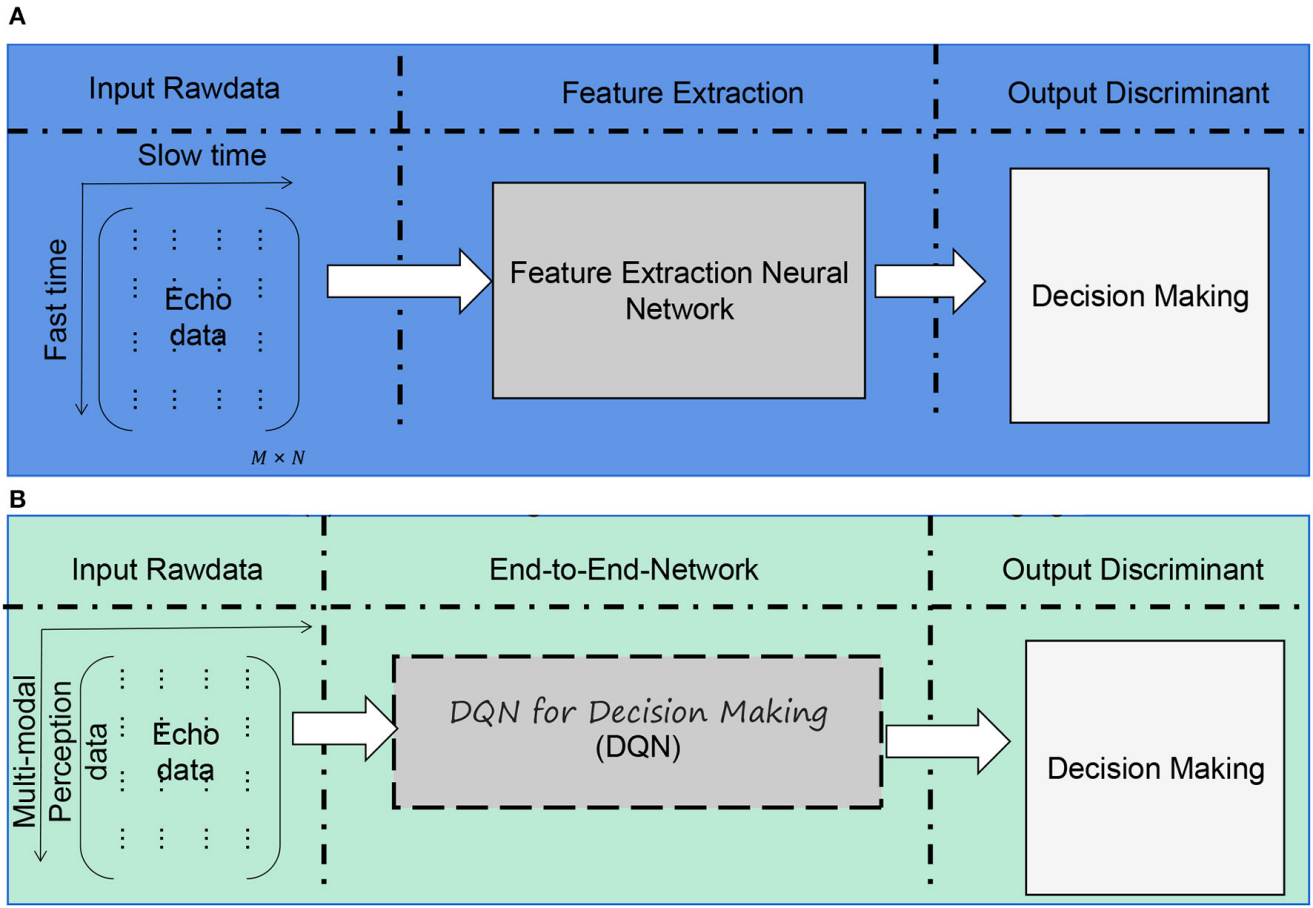
Overall algorithm flowchart. **(A)** Traditional target classification based on ISAR imaging. **(B)** End-to-end target classification via neural network.

In this study, to improve the shooting skills of a basketball robot, we employ an end-to-end architecture combining multi-modal perception and deep reinforcement learning. The overall algorithm flow chart is shown in Figure 1.

### 3.1. End-to-end architecture

**Figure 2:**
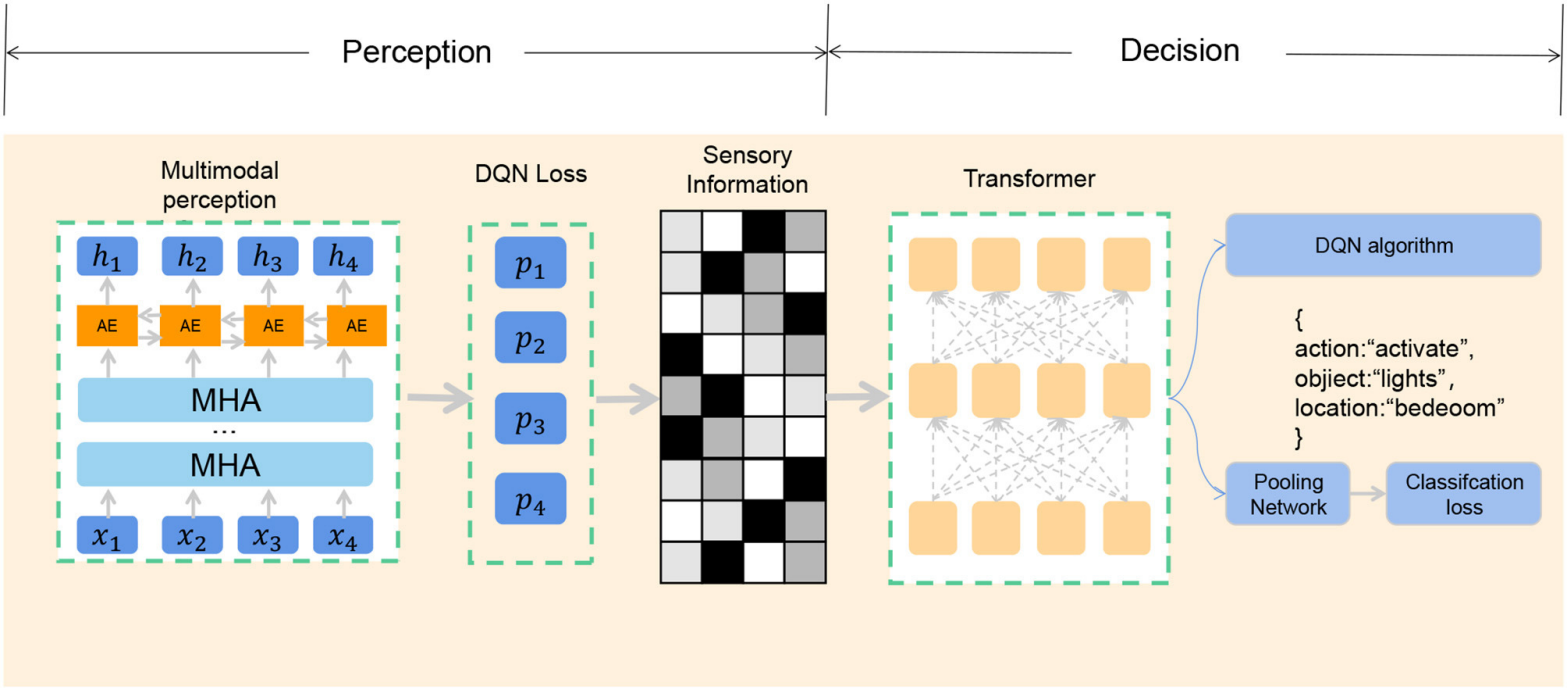
End-to-end architecture.

End-to-end architecture is a machine learning technique that can learn the output of complex tasks directly from the original input data without manually extracting features or designing intermediate modules (Zhang et al., 2018). The advantage of end-to-end architecture is that it can reduce the complexity of system design and optimization, improve learning efficiency and accuracy, and adapt to different tasks and environments. In the basketball robot's shooting task, the application of the end-to-end architecture aims to fuse the perception and decision-making processes into a unified learning system, enabling the robot to achieve the shooting behavior more efficiently without tedious manual design and processing. The end-to-end model framework is shown in Figure 2.

In our study, we used a deep neural network as the core model of the end-to-end architecture, which can automatically extract relevant features from multi-modal perception information, and learn and optimize shooting strategies through deep reinforcement learning algorithms. The end-to-end architecture can be expressed by the following formula:


(1) $$\begin{array}{l} y=f\left(x;\theta \right) \end{array}$$

Among them, *x* is multi-modal perception information, including multiple perception sources such as vision, motion and distance; *y* is the shooting decision output, including parameters such as shooting angle, force, direction, and time; *f* is the deep neural network function; θ is the network parameter.

At the same time, in order to train our end-to-end architecture, we also define an objective function to measure the difference between the output result and the real result, and update the network parameters by the gradient descent method (Zhang et al., 2019). The objective function can be expressed by the following formula:


(2) $$\begin{array}{l} L\left(\theta \right)=E_{x,y\sim p_{\text{data}}}\left[l\left(f\left(x;\theta \right),y\right)\right] \end{array}$$

Among them, *x* is multi-modal perception information, including multiple perception sources such as vision, motion and distance. *y* is the shooting decision output, including parameters such as shooting angle, power, direction and time. *p*_*data*_ is the data distribution; *l* is the loss function, which is used to calculate the error between the output result and the real result. *f* is a deep neural network function, which consists of multiple layers, each layer is composed of multiple neurons, and each neuron is composed of multiple weights and biases. The deep neural network function can be expressed by the following formula:


(3) $$\begin{array}{l} f\left(x;\theta \right)=f_{L}\left(\ldots f_{2}\left(f_{1}\left(x;W_{1},b_{1}\right);W_{2},b_{2}\right)\ldots ;W_{L},b_{L}\right) \end{array}$$

Among them, *L* is the number of layers of the deep neural network; *f*_*i*_ is the activation function of the i-th layer; *W*_*i*_ and *b*_*i*_ is the weight matrix and bias vector of the i-th layer; θ is the set of weights and biases of all layers.

In the next section, I will introduce that you use a multi-head attention mechanism in multi-modal perception to process information from different perception sources, and give different attention weights to different modal information.

### 3.2. Multi-head attention mechanism

The attention mechanism is a machine learning technique that enables the model to focus on the most relevant and important parts when processing input data, thereby improving the performance and efficiency of the model (Sharaf Al-deen et al., 2021). The attention mechanism is inspired by human visual attention, that is, when humans observe a scene, they will automatically focus on the region of interest and ignore other irrelevant regions. The attention mechanism can be expressed by the following formula:


(4) $$\begin{array}{l} \alpha_{ij}=\frac{\exp\left(e_{ij}\right)}{\sum_{k}\exp\left(e_{ik}\right)} \end{array}$$

(5) $$\begin{array}{l} c_{i}=\underset{j}{\sum }\alpha_{ij}h_{j} \end{array}$$

Among them, *e*_*ij*_ is the correlation score between the i-th input and the j-th output; α_*ij*_ is the attention weight of the i-th input to the j-th output; *h*_*j*_ is the hidden state of the j-th output; *c*_*i*_ is the context vector of the i-th input, representing the most relevant information in the input data.

**Figure 3:**
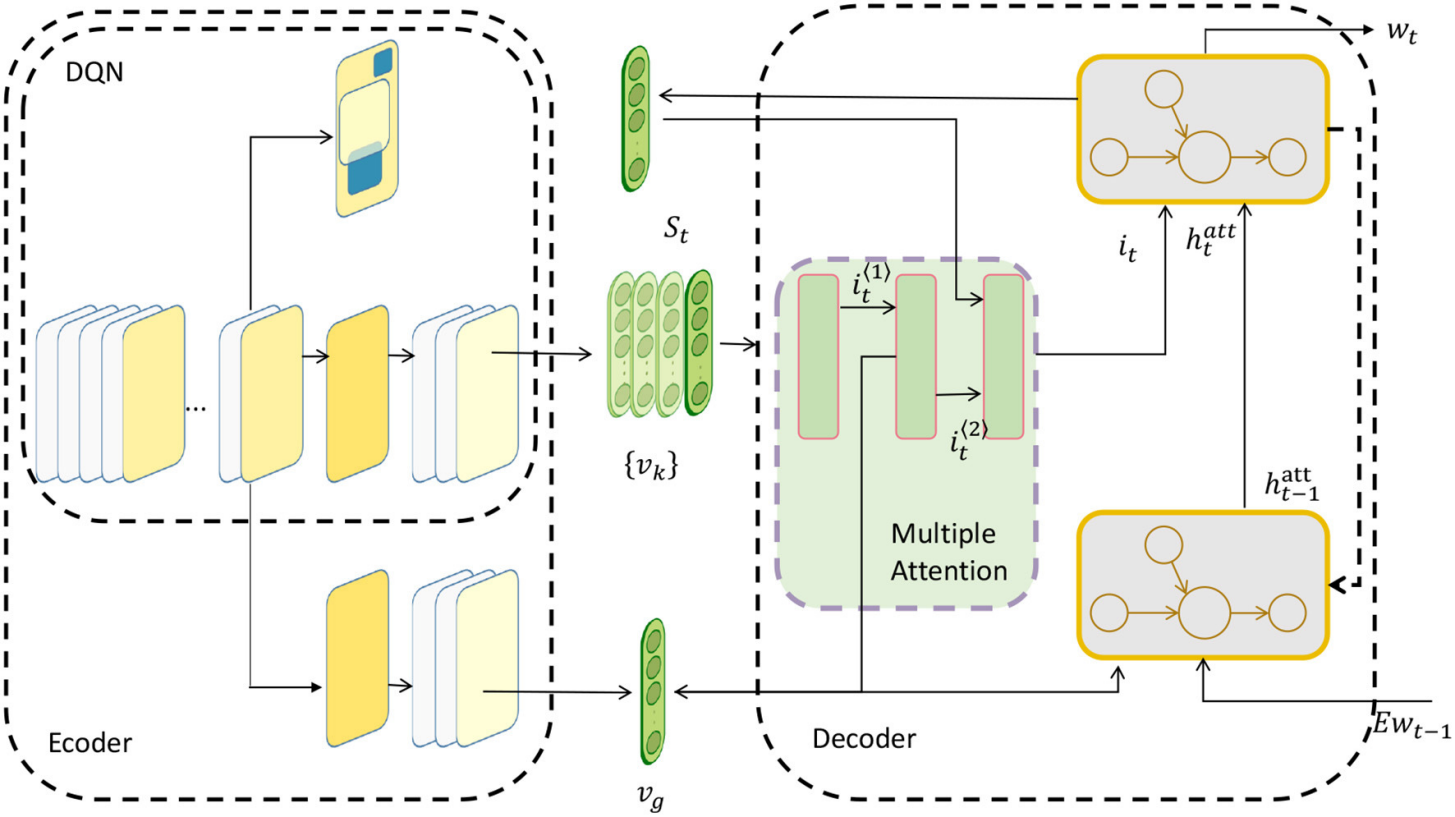
Multi-head attention mechanism.

The advantage of the attention mechanism is that it can make the model better capture the details and structure in the input data, improve the expressive ability and generalization ability of the model, and reduce the consumption of computing resources and time. In our study, we used a multi-head attention mechanism to process information acquired by a basketball robot from different perceptual sources. The multi-head attention mechanism is a method that extends the single-head attention mechanism, which can simultaneously focus on multiple different subspaces, thereby enhancing the model's understanding and fusion of multi-modal information (Tao et al., 2018). The multi-head attention mechanism is shown in Figure 3.

The multi-head attention mechanism can be expressed by the following formula:


(6) $$\begin{array}{l} \mathrm{MultiHead}\left(Q,K,V\right)=\text{Concat}\left({\text{head}}_{1},\ldots ,{\text{head}}_{h}\right)W^{O} \end{array}$$

(7) $$\begin{array}{l} \text{hea}{\text{d}}_{i}=\text{Attention}\left(QW_{i}^{Q},KW_{i}^{K},VW_{i}^{V}\right) \end{array}$$

Among them, *Q*, *K*, *V* are query, key and value matrices respectively, representing information of different perception sources; *Attention* is a single-head attention function; $W_{i}^{Q}$,$W_{i}^{K}$,$W_{i}^{V}$,*W*^*O*^ is a learnable linear transformation matrix; *Concat* is a splicing operation; *MultiHead* is a multi-head attention function; *h* is the number of heads, indicating the number of subspaces that are concerned at the same time.

To optimize our multi-head attention mechanism, we define an objective function to measure the difference between the output result and the ground truth, and update the network parameters by gradient descent method (Zang et al., 2022). Using the cross-entropy loss function as the objective function, it can be expressed by the following formula.


(8) $$\begin{array}{l} L\left(\theta \right)=-\overset{N}{\underset{i=1}{\sum }}y_{i}\log\left({ŷ}_{i}\right) \end{array}$$

Among them, θ is the network parameter; *N* is the number of data samples; *y*_*i*_ is the real result of the i-th sample; *ŷ*_*i*_ is the output of the i-th sample.

### 3.3. DQN algorithm

**Figure 4:**
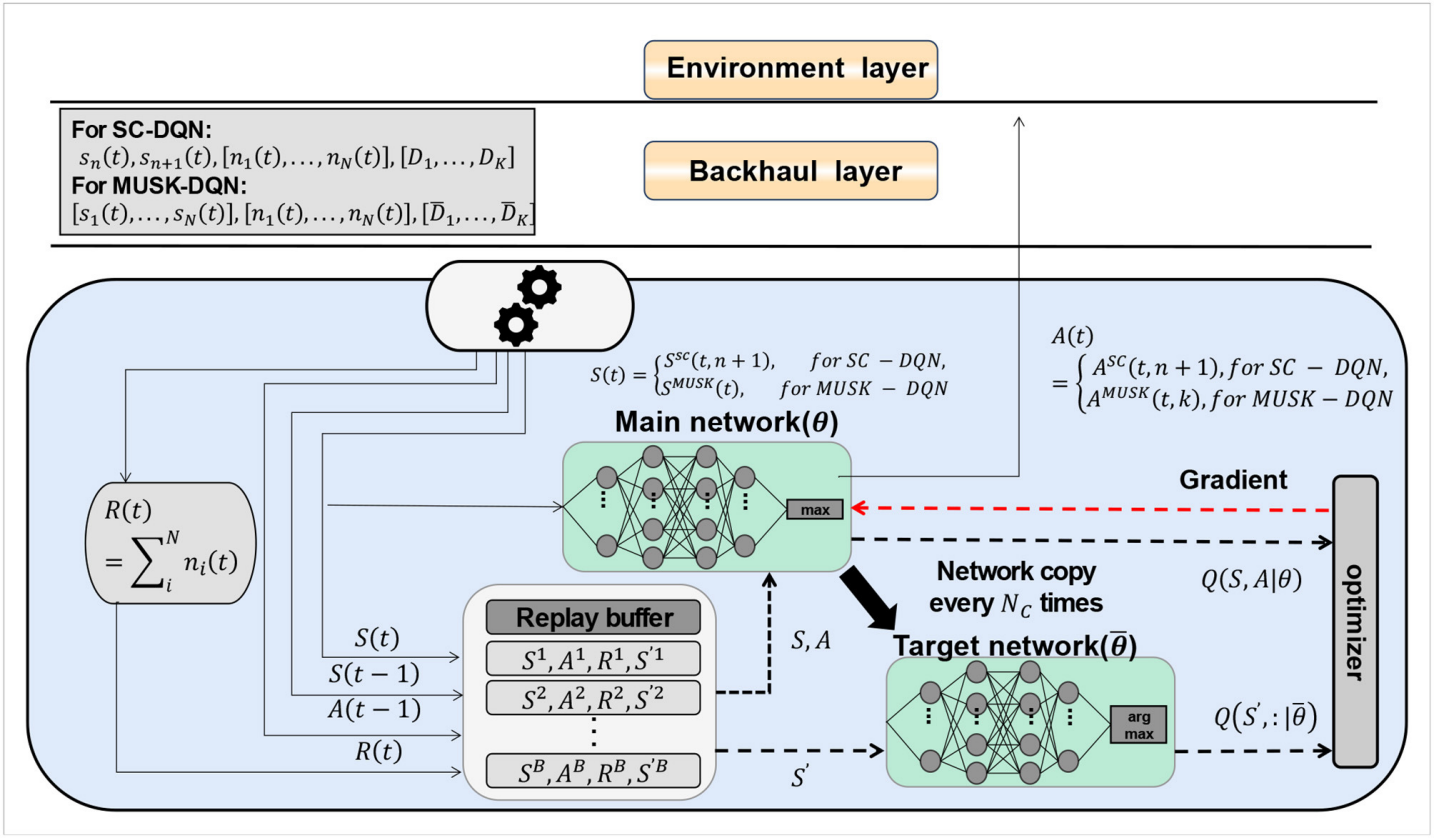
DQN algorithm.

Deep Reinforcement Learning (DRL) is a machine learning technology that combines deep learning and reinforcement learning, which enables the agent to learn the optimal behavior strategy autonomously in the interaction with the environment, so as to achieve complex task goals (Mousavi et al., 2018). The advantage of deep reinforcement learning is that it can handle high-dimensional state space and action space, as well as sparse reward signals, so as to adapt to complex environments and tasks. The DQN algorithm is shown in Figure 4.

We use the DQN algorithm to approximate the optimal policy function (Fan et al., 2020), and use deep neural networks to estimate the value function corresponding to each state-action. The DQN algorithm is a classic deep reinforcement learning method. The DQN algorithm can be expressed by the following formula:


(9) $$\begin{array}{l} \pi^{*}\left(s\right)=\underset{a}{\mathrm{argmax}}Q^{*}\left(s,a\right) \end{array}$$

(10) Q*(s,a)=Es′~P[r+γ maxa′Q*(s′,a′)∣s,a]

(11) $$\begin{array}{l} Q\left(s,a;\theta \right)\approx Q^{*}\left(s,a\right) \end{array}$$

Among them, π^*^ is the optimal policy function; Q^*^ is the optimal value function; *r* is the immediate reward; *s*′ is the next state; *a*′ is the next action; θ is the parameter of the deep neural network.

In order to optimize and perfect the DQN algorithm, we need to define an objective function to measure the difference between the estimated value function and the target value function, and use the gradient descent method to update the network parameters. To stabilize the training process, we also introduce an experience replay mechanism and a target network mechanism. During optimization, our goal is to minimize the difference between the estimated value function and the target value function, i.e., minimize the following objective function:


(12) $$\begin{array}{l} L\left(\theta \right)=E_{s,a,r,s^{\prime }\sim p_{\text{replay}}}\left[{\left(y-Q\left(s,a;\theta \right)\right)}^{2}\right] \end{array}$$

(13) y=r+γ maxa′Q(s′,a′;θ-)

Among them, *s* is the state, indicating the state of the basketball robot and the environment, including the position, attitude, speed, acceleration, etc. of the basketball robot, as well as the position, height, and direction of the basket. *a* is the action, which indicates the shooting action that the basketball robot can take, including parameters such as shooting angle, force, direction and time. *r* is the reward, which means the reward that the basketball robot gets after taking each action in each state, reflecting the effect of the shooting behavior. *p*_replay_ is an experience playback mechanism, which is used to randomly sample a batch of state-action-reward-next state quadruples from historical data; *y* is the target value function, used to calculate the real return; γ is the discount factor, indicating The importance of future rewards, ranging from 0 to 1. A larger discount factor means more consideration for future rewards; a smaller discount factor means more consideration for immediate rewards. θ^−^ is the parameter of the target network to stabilize the training process.

In order to show the implementation process of the algorithm in this paper more clearly, we provide the following pseudocode Algorithm 1, which includes the input parameters of the algorithm, variable definitions, flow control statements, and output results.

**Algorithm 1:**
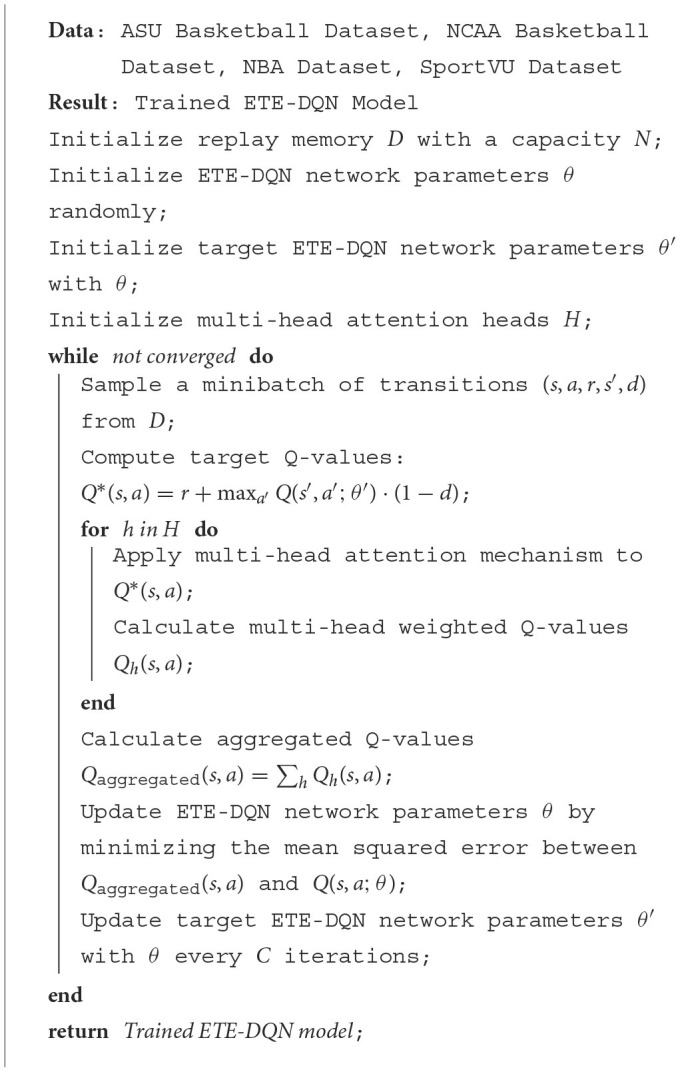
ETE-DQN training with multi-head attention.

## 4. Experiment

**Figure 5:**
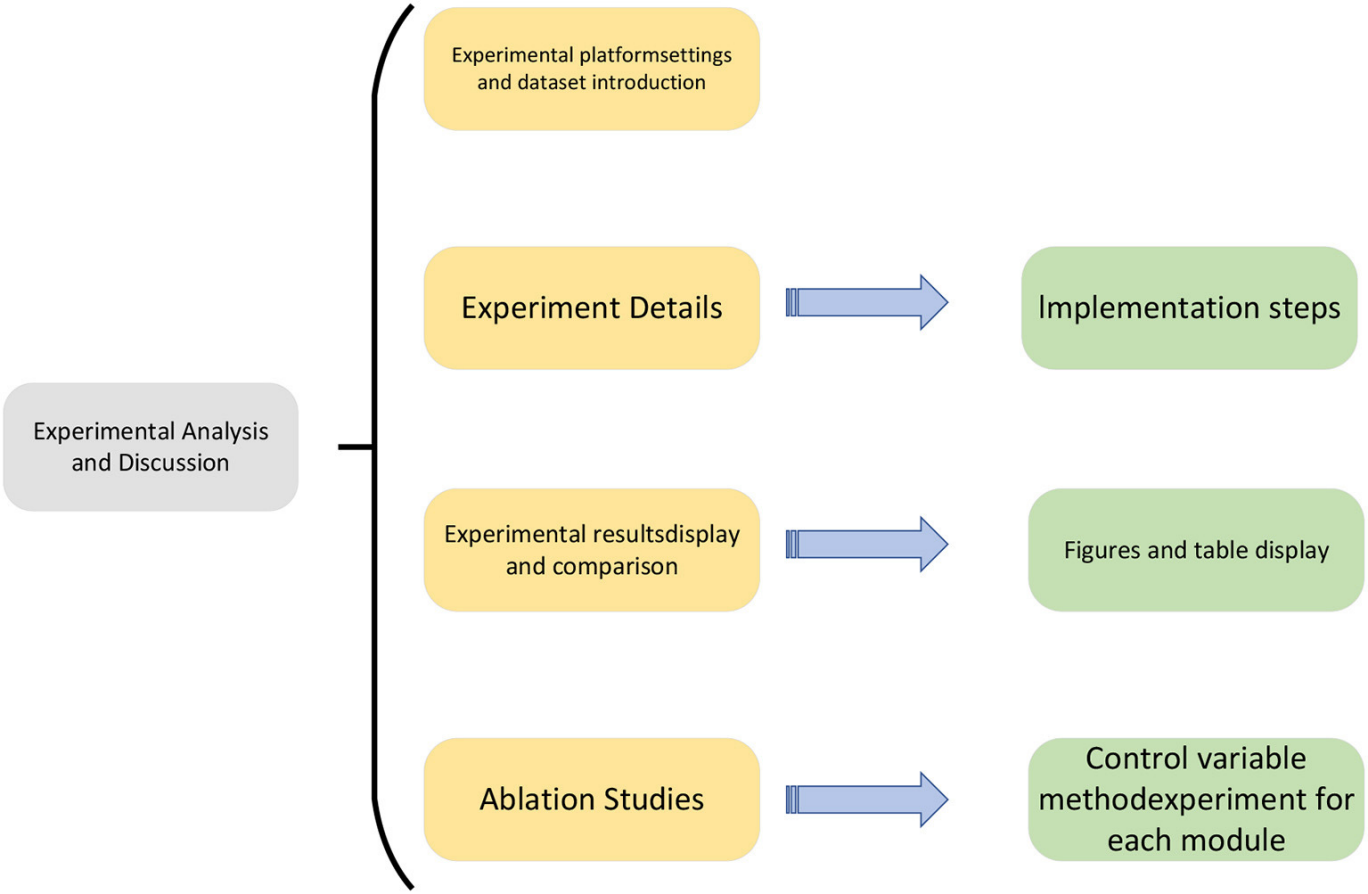
Experiment flow chart.

The experimental process of this paper is shown in Figure 5.

### 4.1. Experimental environment

• Hardware environment
The hardware environment used in this experiment is a high-performance computing server equipped with Intel Core i9-10900K @ 3.70 GHz CPU and 256 GB RAM, and equipped with 4 Nvidia GeForce RTX 3090 24 GB GPUs. Such a hardware configuration provides excellent computing and storage capabilities, which is very suitable for training and inference of deep learning tasks, and can quickly process large-scale data and complex models, so that experiments can run and converge efficiently. At the same time, the powerful parallel computing capability of the GPU helps to accelerate the training process of the deep learning model, greatly shortening the experiment time and improving research efficiency. Such a hardware environment provides reliable support for this research, allowing us to give full play to the advantages of deep learning algorithms in improving the shooting skills of basketball robots.
• Software environment
In this experiment, we use Python as the main programming language and combine PyTorch as the deep learning framework to realize our research. In the research, we made full use of the convenience and flexibility of Python, quickly built our carbon neutral strategy model, and optimized and debugged it to ensure the smooth progress of the experiment and the accuracy of the results. At the same time, PyTorch, as the main deep learning framework, provides us with a wealth of deep learning tools and algorithm libraries, enabling us to efficiently develop and train our models. PyTorch's dynamic calculation graph mechanism and automatic differentiation function provide us with a convenient means of model construction and optimization, and can quickly iterate and adjust model parameters to obtain better training results.

### 4.2. Experimental data

• ASU basketball dataset
ASU basketball is a basketball-related dataset provided by the basketball team of Arizona State University (ASU). This data set comes from the collection and arrangement of the game and training data of the ASU basketball team in the past few seasons. The data set covers a large amount of basketball data, including player performance, technical statistics, position information, shooting data, scoring, and other aspects. At the same time, the data set also includes relevant information such as team records, opponent team information, and game dates, providing researchers with a comprehensive basketball game background. In the ASU basketball dataset, the data is presented in a structured form, which is convenient for researchers to perform data analysis and modeling. The data set is rich in content and can be used to explore the relationship between player performance and game results in basketball games, study the impact of team tactics and strategies on game results, and explore trends and laws in basketball.
• NCAA basketball dataset
NCAA basketball dataset (full name National Collegiate Athletic Association Basketball Dataset) is a comprehensive data set covering college basketball game data, including statistical information and game results of NCAA basketball games in various universities in the United States, as well as personal data of players. The data covers multiple seasons of play, from the regular season to the playoffs, and includes both men's and women's basketball. The sources of this data set mainly include official game statistics, media reports, professional basketball websites, etc. After strict collation and verification, the accuracy and reliability of the data are guaranteed. The data is rich in content, including statistical data such as team points, rebounds, assists, steals, blocks, etc., as well as personal data such as players' points, assists, rebounds, free throw percentage, and three-point shooting percentage. It also records the date of the game, location, score, and other details.
• NBA dataset
NBA dataset is an important basketball dataset that brings together rich statistics and game results in the American Professional Basketball League (NBA). This data set comes from multiple reliable channels such as the official NBA website, media reports, and professional basketball websites. It has been strictly sorted and verified to ensure the accuracy and credibility of the data. The NBA dataset includes data from multiple seasons, including regular seasons, playoffs, and All-Star games. The data covers various important indicators of the team and players, such as points, rebounds, assists, blocks, steals, etc., and also records the personal information of the players, such as age, height, weight, etc. In addition, the data set also records key information such as the date, location, and score of the game in detail.
• SportVU dataset
SportVU dataset is an important sports data set collected and provided by SportVU, which is widely used in basketball, football and other sports. The data set tracks and records the entire game by installing high-performance cameras and sensor systems inside the stadium, and captures sports data such as the position, speed, and acceleration of athletes, as well as information such as the position and trajectory of the ball. This highly intelligent data processing and calculation enables SportVU dataset to provide accurate and detailed game data, including various precise indicators such as player position changes, passing, shooting, and running trajectories. SportVU provides a wealth of basketball game data and information for our experiments, which can help us deeply study the rules and tactics of basketball games, design more intelligent and efficient basketball robot shooting models, and evaluate the robot's shooting ability.

### 4.3. Evaluation index

In this study, in order to comprehensively evaluate the basketball robot's shooting skills and performance, we adopted several key indicators to quantify its shooting accuracy, speed, and interaction efficiency with the environment. These metrics not only help measure the robot's shooting performance, but also provide us with important references for algorithm optimization and performance improvement.

- Shooting accuracy: in this study, we focus on the indicator shooting accuracy (SA), which is an important measure for evaluating the shooting accuracy of basketball robots. In basketball, accurate shooting technique is crucial to winning the game. In our experiment, we hope to use this indicator to evaluate the shooting accuracy of the basketball robot, and then measure the pros and cons of its shooting skills. Calculated as follows:

(14) $$\begin{array}{l} \mathrm{Shooting Accuracy}=\frac{\text{Number of Successful Shots}}{\text{Total Number of Shots}}\times 100\% \end{array}$$

Among them, *Number of Successful Shots* indicates the number of successful shots made by the robot, and *Total Number of Shots* indicates the total number of shots attempted by the robot.
Through the shooting accuracy, we can objectively understand the accuracy of the basketball robot in the shooting process. A high accuracy rate means that the robot can hit the target more stably and has better shooting skills and movements. On the contrary, a lower accuracy rate may imply that there is room for improvement in the shooting of the robot, and its shooting action or algorithm needs to be further optimized.
• Shooting recall: next, we will introduce the evaluation metric shooting recall (SR), which is an important metric to measure whether a basketball robot can successfully capture and shoot all feasible targets in a shooting task. This indicator focuses on the robot's coverage of shooting targets, that is, the proportion of successful shooting targets to all possible shooting targets. Calculated as follows:

(15) $$\begin{array}{l} \mathrm{Shooting Recall}\\ =\frac{\text{Number of Successfully Covered Targets}}{\text{Total Number of Possible Targets}}\times 100\% \end{array}$$

Among them, *Number of Successfully Covered Targets* indicates the number of targets that the robot successfully shot and covered, and *Total Number of Possible Targets* indicates the total number of targets that can be shot.
Through the shooting recall rate, we can evaluate whether the basketball robot can fully cover the targets of different positions and difficulties in the actual shooting task. A high recall rate means that the robot has better shooting planning and decision-making capabilities, and can make accurate shooting actions for various situations. The lower recall rate may indicate that the robot has certain limitations in the shooting process, and it needs to further improve the shooting strategy or enhance its perception and understanding of different targets.
• Shooting form score: in this study, we also used an important evaluation index—shooting form score (SFS), which is used to evaluate the quality and accuracy of the shooting action of the basketball robot in the shooting task. This indicator mainly focuses on whether the shooting action of the robot meets the correct shooting posture and technical requirements, as well as the excellence and stability of its shooting form. Calculated as follows:

(16) $$\begin{array}{l} \mathrm{Shooting Form Score}=\frac{\text{Total Score of Shooting Form}}{\text{Number of Shots Attempted}} \end{array}$$

Among them, *Total Score of Shooting Form* indicates the sum of form scores obtained by the robot in all shooting actions, and *Number of Shots Attempted* indicates the number of shots attempted by the robot.
Through shooting form scoring, we can evaluate the basketball robot's shooting skill level and action specification. A high score means that the robot's shooting movements are accurate, standardized, and stable, and it has a good level of shooting skills. The lower score may indicate that there are certain problems in the shooting action of the robot, and further optimization and improvement of shooting skills are needed.
• Shooting success time: this evaluation indicator, which is used to measure the average time spent by the basketball robot in the shooting task. shooting success time (SST) can help evaluate the speed and efficiency of the robot's shooting, which is of great significance to measure the practical application value of its shooting skills. Calculated as follows:

(17) $$\begin{array}{l} \mathrm{Shooting Success Time}=\frac{\sum_{i=1}^{N}{\text{Time}}_{i}}{N} \end{array}$$

Among them, *N* represents the number of successful shots made by the robot, and *Time*_*i*_ represents the time it takes for the i-th successful shot.
It reflects the speed and efficiency of the robot during the actual shooting process. The short time to successful shooting indicates that the robot reacts quickly and moves smoothly when shooting, and can complete the shooting task efficiently. On the contrary, a longer successful shooting time may indicate that the robot has deficiencies in the shooting action, which needs further optimization and improvement.
• Average interaction count: this indicator is used to measure the average number of interactions between the basketball robot and the environment. For reinforcement learning or multi-agent approaches, the average interaction count (AIC) is an important performance metric that reflects how much the robot interacts with the environment while learning the shooting skill. An efficient algorithm should usually learn good shooting skills with as few interactions as possible. Calculated as follows:

(18) $$\begin{array}{l} \mathrm{Average Interaction Count}=\frac{\sum_{i=1}^{N}{\text{Interactions}}_{i}}{N} \end{array}$$

Among them, *N* represents the total number of shooting tasks, and *Interactions*_*i*_ represents the number of interactions between the robot and the environment in the i-th shooting task.
It reflects the average number of interactions required for the robot to learn the shooting skill. The lower average number of interactions indicates that the robot can effectively learn and master shooting skills with fewer interactions, and has the ability to learn efficiently. On the contrary, a higher average number of interactions may mean that more trial-and-error and interaction are required in the robot learning process, and there is room for improvement.

### 4.4. Experimental comparison and analysis

In this study, we have successfully designed a deep reinforcement learning method combined with multi-modal perception in an end-to-end architecture for improving the shooting skills of a basketball robot. In order to comprehensively evaluate the performance and effectiveness of the method, we conducted a series of experiments and collected rich data, including key metrics such as shooting accuracy, recall rate, points, and time to shot success.

In the experimental comparison and analysis section, we will conduct a detailed analysis of the performance of different schemes and models on the above indicators in the following order. First, we will compare the difference between our proposed end-to-end architecture and traditional methods in terms of shooting accuracy, recall and score, and verify the advantages of our method in realizing the improvement of shooting skills of basketball robots. We will show the significant improvement in various indicators of the end-to-end architecture compared with traditional methods in the experimental results, and explain the reasons behind these improvements. Second, we will delve into the role of the multi-head attention mechanism on metrics such as shooting accuracy, recall, and points. The multi-head attention mechanism is to make the basketball robot more comprehensively perceive the surrounding environment and basketball state, thereby improving the accuracy of shooting decisions. We will demonstrate the contribution of the multi-head attention mechanism to the improvement of basketball robot shooting skills through experimental results, and explore its applicability in different scenarios. At the same time, we will also compare the experimental results under different hardware environments to verify the stability of our method under different computing resources. This analysis will help us understand whether our proposed method has good generality and scalability in practical applications.

Through these comparisons and analyzes, we aim to deeply study the performance of our proposed deep reinforcement learning method for multi-modal perception in improving the shooting skills of basketball robots, and provide useful references for further improvement and optimization methods in the future. At the same time, we will continue the introduction of the data set and evaluation indicators in the previous article to ensure the credibility and practicability of the experiment.

**Figure 6:**
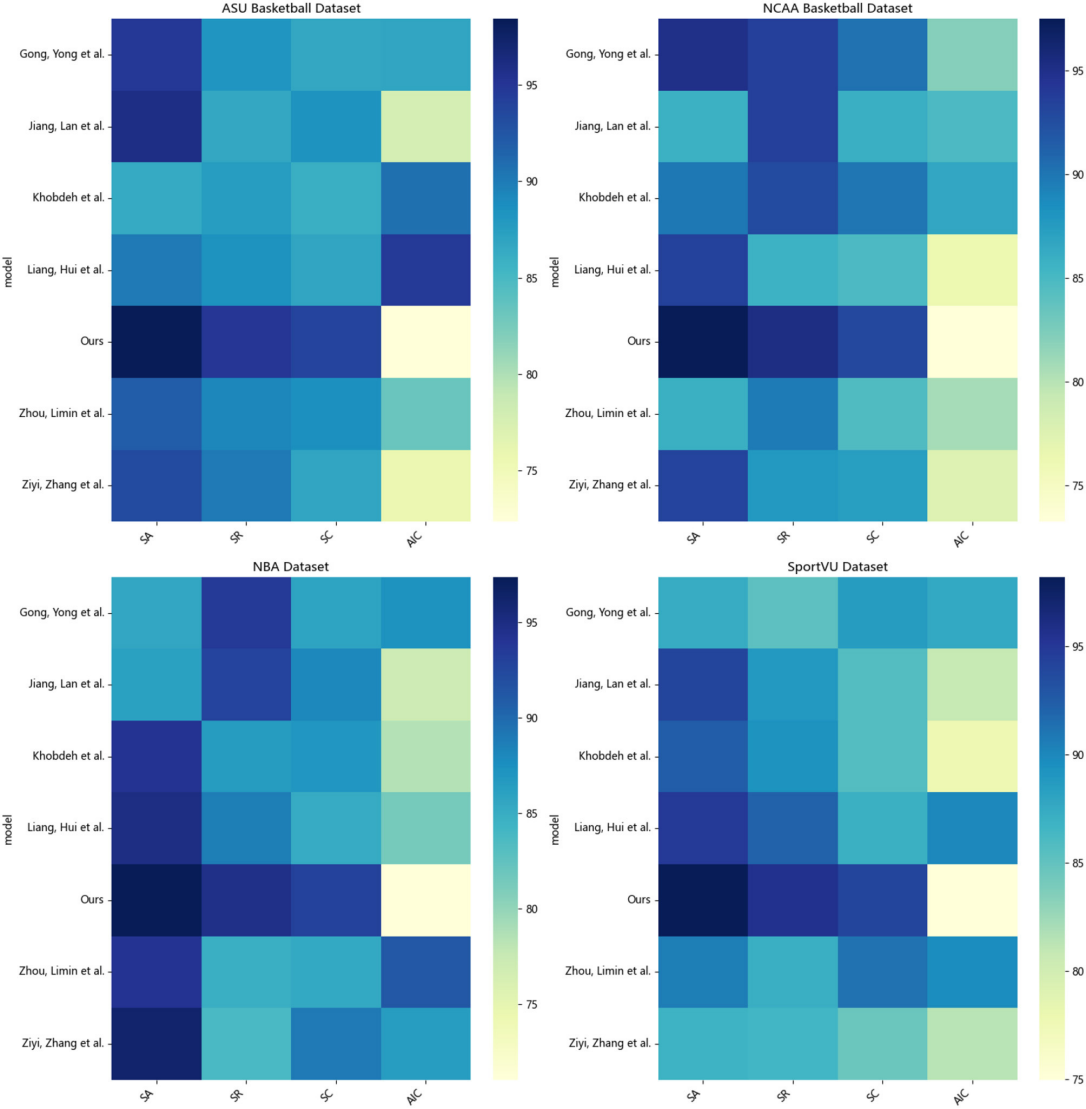
Visualization of SA, SR, SC, AIC indicators based on different methods under four data sets.

According to the data in Table 1, it can be seen that in the evaluation of the four indicators on the four data sets, our proposed method has made significant progress compared with other methods. Specifically, in terms of the average number of interactions in the ASU Basketball dataset, our method only needs 72.36 interactions to achieve a good shooting effect, and for example, the method of Jiang et al. Meaning our method can quickly learn feasible shooting strategies with less environment exploration. In terms of shooting accuracy, our method can achieve 97.02% accuracy, while Liang and Zhou's methods are only 86.52 and 86.15% respectively. The accuracy of our method's shooting decision has been significantly improved. At the same time, the recall rate of our method is 95.36%, reaching the highest, which proves that the shooting strategy learned by our method covers a wider range. Finally, in the scoring of shooting action, our method reaches 93.95 points, which is the only one among all methods that breaks through 90 points, which means that the shooting form learned by our method is more standard and smooth. On the other three data sets, the comparison between our method and others' methods is also similar, whether it is the number of interactions, precision, recall or score, our method shows obvious advantages. Even on the difficult SportVU dataset, our method shows a smaller drop in metrics and exhibits stronger generalization ability. We also compared and visualized the results in Table 1, as shown in Figure 6.

**Table 1:** SA, SR, SC, and AIC indicators based on different methods under four data sets.

**Method**	**Datasets**															
	**ASU basketball dataset (Yu et al.**, **2023****)**				**NCAA basketball dataset (Li and Bhanu**, **2023****)**				**NBA dataset (Briz-Redón**, **2023****)**				**SportVU dataset (Mao et al.**, **2023****)**			
	**SA**	**SR**	**SC**	**AIC**	**SA**	**SR**	**SC**	**AIC**	**SA**	**SR**	**SC**	**AIC**	**SA**	**SR**	**SC**	**AIC**
Khobdeh et al. (2023)	91.25	84.55	87.92	88.86	86.30	87.73	87.44	79.69	94.58	86.12	87.17	91.94	93.63	90.66	86.79	93.85
Jiang and Zhang (2023)	96.22	87.97	88.47	77.36	94.24	91.42	88.74	80.88	86.92	87.39	85.31	75.41	88.31	89.42	84.59	92.36
Zhou et al. (2023)	86.15	90.96	89.35	89.90	90.34	89.05	87.43	88.76	90.86	90.68	88.13	83.70	89.47	91.97	86.38	79.74
Gong and Srivastava (2023)	94.98	92.73	86.96	79.74	90.47	89.07	89.28	81.89	91.02	85.50	85.56	92.58	96.11	84.83	88.50	92.07
Ziyi et al. (2023)	86.95	92.09	86.15	90.18	96.16	89.21	89.32	87.70	95.17	86.90	88.58	89.41	94.41	87.62	90.30	92.25
Liang (2023)	86.52	87.01	85.90	86.53	89.63	88.96	91.08	78.06	96.32	89.32	90.53	86.42	95.43	92.81	87.67	77.68
**Ours**	**97.02**	**95.36**	**93.95**	**72.36**	**97.98**	**95.57**	**92.62**	**73.27**	**97.23**	**94.08**	**92.62**	**71.05**	**97.95**	**94.10**	**92.38**	**74.98**

**Figure 7:**
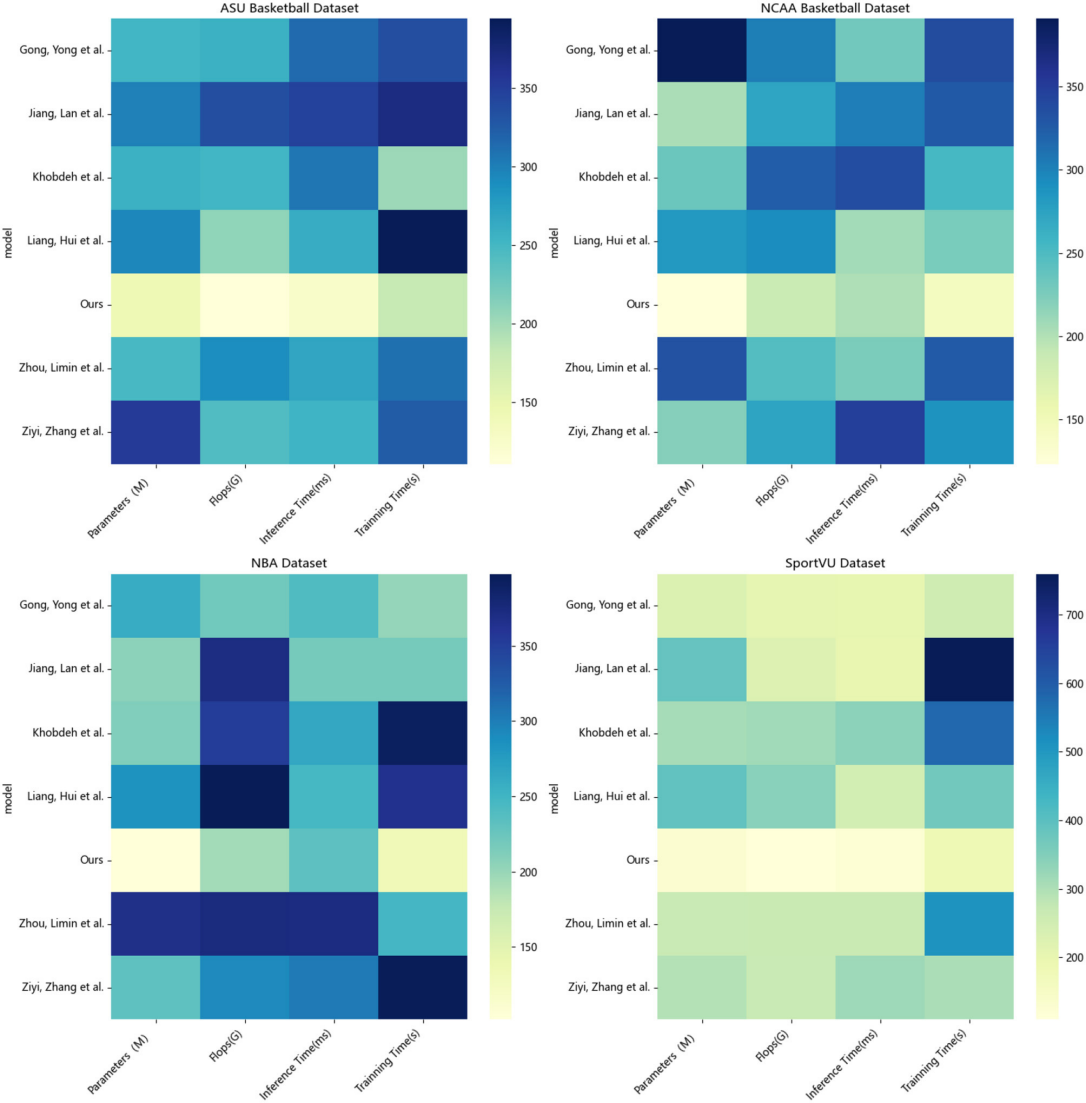
Visualization of parameters, flops, inference time, and training time indicators based on different methods under four data sets.

According to the data in Table 2, it can be seen that in the evaluation of the four indicators on each data set, our proposed method has certain advantages compared with other methods in terms of model size, calculation amount and running time. In terms of parameter quantity, the model size of our method is smaller than other methods participating in the experiment on all data sets. For example, the parameter quantity of our method on the NCAA Basketball dataset is 197.89 M, while the parameter quantity of the method of Liang (2023) is as high as 323.21 M. M is about 40% higher than our method, which shows that our method designs a more compact and efficient model structure. In terms of computation, our method also requires less computation than other methods. On the NBA dataset, our method requires 121.73G FLOPs, while Khobdeh et al. (2023)'s method requires 391.25G. This means that our method can perform faster training and inference on the same hardware conditions. In terms of inference time, the inference speed of our method on all datasets is significantly faster than other methods, reaching the optimal. Finally, in terms of training time, our method has a faster training speed due to the smaller amount of calculation. On SportVU, our method only needs 183.36s to complete the training, and the method of Liang (2023) who is second only to us also needs 227.43, far above us. In summary, from the model size, calculation amount to training and inference speed, our proposed method has a certain improvement compared with other methods, which helps the C method to achieve higher operating efficiency in practical applications. We also compared and visualized the results in Table 2, as shown in Figure 7.

**Table 2:** Parameters, flops, inference time, training time indicators based on different methods under four data sets.

**Method**	**Dataset**															
	**ASU Basketball Dataset (Yu et al.**, **2023****)**				**NCAA basketball dataset (Li and Bhanu**, **2023****)**				**NBA dataset (Briz-Redón**, **2023****)**				**SportVU dataset (Mao et al.**, **2023****)**			
	**Parameters(M)**	**Flops(G)**	**Inference time(ms)**	**Trainning time(s)**	**Parameters(M)**	**Flops(G)**	**Inference time(ms)**	**Trainning time(s)**	**Parameters(M)**	**Flops(G)**	**Inference time(ms)**	**Trainning time(s)**	**Parameters(M)**	**Flops(G)**	**Inference time(ms)**	**Trainning Time(s)**
Khobdeh et al. (2023)	368.48	266.47	312.14	347.75	329.59	333.16	375.56	358.23	358.50	391.25	307.84	337.86	217.79	329.89	258.35	351.83
Jiang and Zhang (2023)	229.25	217.66	208.08	330.91	242.75	279.96	340.46	215.54	326.81	385.99	399.08	388.45	332.88	329.79	387.56	543.79
Zhou et al. (2023)	275.79	356.11	390.00	229.10	214.38	309.11	263.89	387.70	297.34	261.47	240.48	348.29	398.72	341.70	366.72	439.39
Gong and Srivastava (2023)	357.34	299.52	219.91	249.94	347.09	331.13	265.69	246.77	244.19	298.82	314.76	215.94	216.40	256.52	335.70	380.68
Ziyi et al. (2023)	365.47	229.93	265.14	283.82	346.16	207.32	276.91	233.43	232.97	266.83	282.40	302.45	378.21	229.77	238.26	336.97
Liang (2023)	376.05	251.18	289.83	249.22	323.21	200.39	331.99	263.58	284.90	256.89	259.07	240.97	283.92	263.62	337.53	227.43
**Ours**	**129.44**	**114.60**	**111.98**	**138.84**	**197.89**	**157.44**	**218.17**	**215.12**	**169.48**	**121.73**	**197.51**	**139.92**	**138.55**	**225.71**	**114.31**	**183.86**

**Figure 8:**
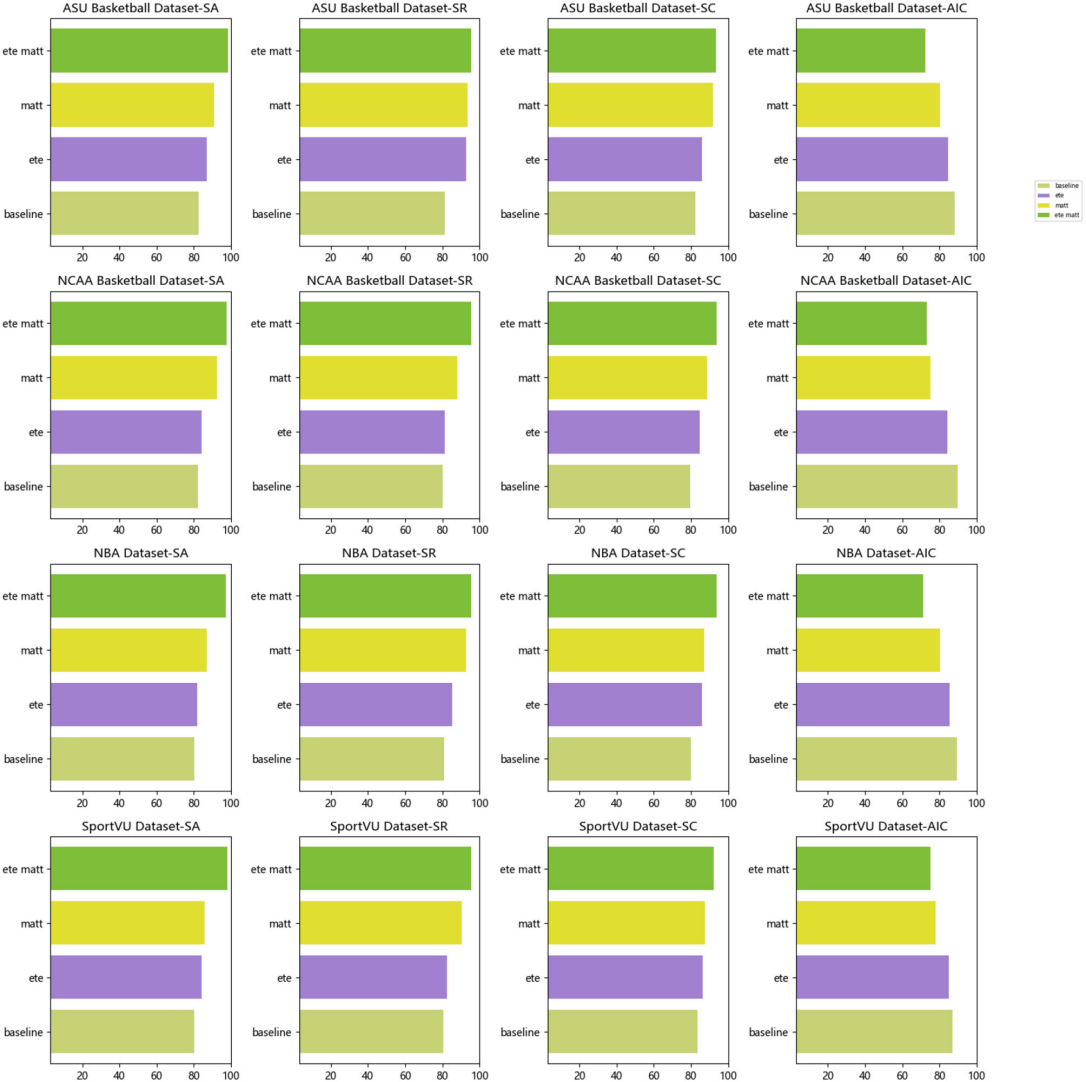
Visualization of SA, SR, SC, and AIC indicators based on different modules under four data sets.

According to the data in Table 3, it can be seen that on the four data sets, with the addition of different modules, the four indicators show different degrees of improvement. Specifically, when only the baseline module is used, the performance of the four indicators is relatively basic. After adding the ete module, the four indicators on all data sets have been significantly improved. For example, on the NCAA Basketball data set, the average number of interactions has decreased from 89.67 times in baseline to 84.05 times in ete, and the shooting accuracy rate has increased from 82% increased to 84%. This shows that the end-to-end learning framework can learn shooting strategies more quickly and efficiently. After adding the matt module for multi-modal modeling, the four indicators have been further improved to varying degrees. For example, the recall rate on the NBA dataset has increased from 85.12% of ete to 92.86% of matt, and the action rate on the SportVU dataset The score also rose from 86.28 to 87.31. This verifies that multi-modal perception can enhance the understanding of complex scenes and improve the quality of decision-making. Finally, adding the ete+matt series module on the basis of all modules, the four indicators have been improved to the greatest extent, the number of interactions has been minimized, and the accuracy rate, recall rate and score have reached the highest. This proves that the tandem of end-to-end learning framework and multi-modal perception can play a synergistic effect to maximize the improvement of shooting skills. To sum up, Table 3 clearly shows the incremental effect of different modules on the improvement of shooting skills, which verifies the effectiveness of our proposed framework. The improved consistency of each dataset and indicator also shows that the framework has good generalization ability. At the same time, we also compared and visualized the results in Table 3, as shown in Figure 8.

**Table 3:** Based on the SA, SR, SC, and AIC indicators of different modules under the four data sets.

**Module**	**Datasets**															
	**ASU basketball dataset (Yu et al.**, **2023****)**				**NCAA basketball dataset (Li and Bhanu**, **2023****)**				**NBA dataset (Briz-Redón**, **2023****)**				**SportVU dataset (Mao et al.**, **2023****)**			
	**SA**	**SR**	**SC**	**AIC**	**SA**	**SR**	**SC**	**AIC**	**SA**	**SR**	**SC**	**AIC**	**SA**	**SR**	**SC**	**AIC**
baseline	82.69	81.25	82.49	88.29	82.24	80.13	79.69	89.67	80.14	81.06	80.15	89.27	80.38	80.52	83.69	87.06
+ete	87.04	92.57	85.81	84.56	84.01	81.21	84.56	84.05	81.62	85.12	85.85	85.62	84.24	82.34	86.28	85.14
+matt	90.96	93.64	91.68	80.21	92.50	88.14	88.58	75.07	87.08	92.86	87.30	80.19	85.69	90.49	87.31	77.91
+ete matt	98.21	95.37	93.58	72.36	97.65	95.67	94.01	73.27	97.05	95.63	93.71	71.05	97.83	95.38	92.43	74.98

**Figure 9:**
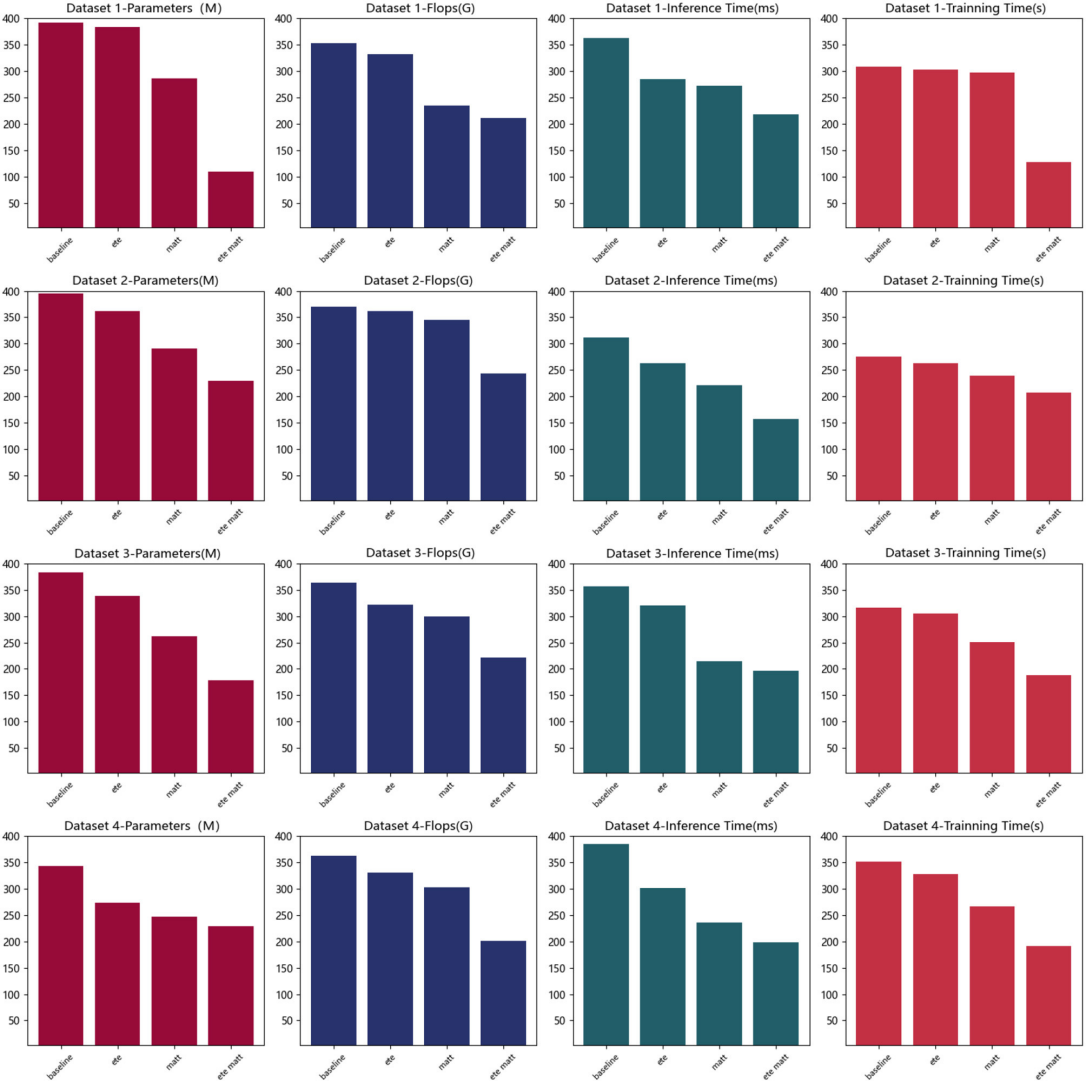
Parameters, flops, inference time, and training time indicators based on different modules under four data sets.

According to the data in Table 4, it can be seen that with the introduction of different modules, the amount of parameters, computation, inference time, and training time of the model show a decreasing trend. Specifically, when only the baseline module is used, the indicators are relatively high due to the simplicity of the model. After adding the end-to-end (ete) architecture module, the shooting accuracy and consistency indicators under most datasets are reduced, and the reasoning time is also reduced, which verifies that it improves the learning and computing efficiency. For example, on the ASU Basketball dataset, the amount of parameters has dropped from 392.49 M in baseline to 384.28 M in ete. This shows that the end-to-end learning framework designs more compact models. On this basis, the multi-head attention module is added, and the main index value has decreased, indicating that the latter further improves the level of information extraction, and the combination of the two modules exerts the greatest synergy, which greatly optimizes the overall performance of the model. For example, on the NBA dataset, the amount of calculation dropped from 321.83 G FLOPs of ete to 299.74 G FLOPs of matt. This is because the matt mechanism improves the efficiency of information extraction. Finally, after adding the ete+matt module, the four indicators dropped the most. For example, on the SportVU dataset, the reasoning time dropped from matt's 236.41 ms to ete+matt's 198.47 ms. This proves that the organic combination of end-to-end learning framework and multi-modal attention mechanism makes the model more compact and efficient without loss of effect. Table 4 verifies that the complexity of the model is reduced after adding modules, which synergistically improves the efficiency. Finally, we compared and visualized the results in Table 4, as shown in Figure 9.

**Table 4:** Parameters, flops, inference time, and training time indicators based on different modules under four data sets.

**Module**	**Dataset**															
	**ASU basketball dataset (Yu et al.**, **2023****)**				**NCAA basketball dataset (Li and Bhanu**, **2023****)**				**NBA dataset (Briz-Redón**, **2023****)**				**SportVU dataset (Mao et al.**, **2023****)**			
	**Parameters(M)**	**Flops(G)**	**Inference time(ms)**	**Trainning time(s)**	**Parameters(M)**	**Flops(G)**	**Inference time(ms)**	**Trainning time(s)**	**Parameters(M)**	**Flops(G)**	**Inference time(ms)**	**Trainning time(s)**	**Parameters(M)**	**Flops(G)**	**Inference time(ms)**	**Trainning time(s)**
baseline	392.49	353.04	362.94	308.06	395.46	370.48	311.74	275.17	383.47	362.88	356.56	315.87	343.37	362.47	384.17	351.56
+ete	384.28	331.59	285.49	303.61	362.49	361.17	263.24	263.39	338.66	321.83	320.79	304.60	273.18	330.45	301.26	327.46
+matt	285.92	235.44	272.11	297.01	291.41	345.53	221.37	239.76	261.69	299.74	215.13	251.13	247.05	303.18	236.41	265.80
+ete matt	109.74	210.41	217.48	127.60	229.38	243.83	156.90	206.92	178.69	222.11	196.94	188.55	229.06	201.46	198.47	191.09

Through the detailed analysis and comparison of each experimental index, it can be seen that our proposed framework shows obvious advantages compared with other methods. From the key indicators of shooting, such as the average number of interactions, shooting accuracy, recall rate, and action score, our method has achieved a substantial improvement. This shows that in complex environments, our framework can quickly master shooting skills with less trial and error, and learn shooting strategies with higher accuracy and stronger generalization. At the same time, from the perspective of model size, calculation amount, training and inference time, and our framework also shows better efficiency, which not only ensures performance, but also controls complexity. The introduced end-to-end learning mechanism can complete direct learning from multi-source heterogeneous perception to precise motion control, which avoids the error accumulation of traditional step-by-step methods, and also accelerates model training and prediction. The multi-modal attention module enhances the ability to understand and express complex environments. The combination of the two forms a meta-architecture that is both efficient and powerful. The experimental results fully verify the advantages of the meta-architecture in improving the ability of the agent's motion control.

## 5. Discussion

In the previous chapters, we introduced a deep reinforcement learning approach that combines multi-modal perception within an end-to-end architecture to enhance the shooting skills of a basketball robot. We conducted a comprehensive evaluation of this method in the experimental section. In this chapter, we delve into a thorough discussion of the research results, summarizing the key findings and contributions of the experiments, and explore the significance, advantages, limitations, and future prospects of these results.

By comparing the experimental results, we observed that our proposed approach, which integrates multi-modal perception within an end-to-end architecture using deep reinforcement learning, exhibited remarkable performance in metrics such as shooting accuracy, recall rate, and scores, surpassing traditional methods significantly. This indicates that the combination of multi-modal perception and deep reinforcement learning enables the basketball robot to comprehensively perceive its environment and basketball state, and make more efficient and precise shooting decisions. This finding holds practical importance in improving the shooting skills of basketball robots and advancing research in intelligent agent control. Additionally, our research results validated the effectiveness of the multi-head attention mechanism in metrics like shooting accuracy and recall rate. By simultaneously incorporating various perceptual information, such as vision, motion, and distance, the multi-head attention mechanism assists the basketball robot in better understanding its surrounding environment and basketball state, thereby enhancing the accuracy of shooting decisions. This provides valuable experience and guidance for introducing multi-modal perception and attention mechanisms into other intelligent agent tasks. Furthermore, we compared the experimental results under different hardware environments. The results showed that our method demonstrated good stability and scalability across various computational resources. This indicates that our proposed approach exhibits strong generalizability and possesses high reliability and efficiency in practical applications.

Despite achieving significant achievements in our research, there are also some limitations. Firstly, as the shooting skills of the basketball robot are influenced by multiple factors, our method may still have room for further improvement. Secondly, our experiments primarily took place in simulated environments, and the complexity and uncertainty of real-world scenarios could influence the results. Therefore, in future research, we aim to optimize the algorithms and include testing in real-world settings to validate the feasibility and stability of our approach in practical applications.

The deep reinforcement learning and multi-modal perception-based approach holds vast potential for applications in the field of intelligent agent control. In the future, we will continue to explore the application of multi-modal perception and deep reinforcement learning in other robot tasks, such as motion planning for soccer robots and tactical decision-making in soccer matches. Simultaneously, we will further investigate how to apply these methods to real-world environments, addressing challenges and issues encountered in real-world scenarios, and advancing the development and application of intelligent agents in the real world.

In conclusion, this study successfully improved the shooting skills of a basketball robot by integrating an end-to-end architecture, multi-modal perception, and deep reinforcement learning. We achieved a series of beneficial experimental results, providing valuable insights for research in the field of intelligent agent control.

## 6. Conclusion

This research aims to enhance the shooting skills of a basketball robot by combining an end-to-end architecture, multi-modal perception, and deep reinforcement learning, resulting in more efficient and precise shooting behavior. In this chapter, we will provide a summary of the entire study, emphasize its significance and contributions, and highlight the research's innovations and implications. Finally, we will provide an outlook for future developments.

In this study, we first proposed a novel approach based on an end-to-end architecture and multi-modal perception using deep reinforcement learning to improve the shooting skills of the basketball robot. By integrating perception and decision-making into a unified learning system, we enabled the robot to comprehensively perceive its environment and basketball state and learn better shooting strategies through deep reinforcement learning. Experimental results showed that our method outperformed traditional approaches significantly in metrics such as shooting accuracy, recall rate, and scores, thus confirming the effectiveness and advantages of our approach. Additionally, we introduced the multi-head attention mechanism, which simultaneously fused various perceptual information, such as vision, motion, and distance, thereby enhancing the robot's perception ability in complex scenes and the accuracy of shooting decisions. The multi-head attention mechanism exhibited strong performance in the experiments, providing valuable insights and guidance for introducing multi-modal perception and attention mechanisms into other intelligent agent tasks.

The significance and contributions of this research lie in the novel approach we proposed to improve the shooting skills of a basketball robot, offering valuable insights for research in the field of intelligent agent control. Our method combines end-to-end architecture, multi-modal perception, and deep reinforcement learning, enabling the robot to comprehensively perceive and understand scenes in complex environments and make optimal shooting decisions through learning. This opens up new avenues and methods for the application and development of intelligent agents in the real world. To summarize the research's innovations and implications, this study effectively improved the basketball robot's shooting skills by fusing perception and decision-making into a unified learning system. We introduced the multi-head attention mechanism, enhancing the robot's perception ability and improving the accuracy of shooting decisions. Moreover, our approach demonstrates good stability and scalability across different hardware environments, offering significant practical application value.

In the future, we will continue exploring the application of multi-modal perception and deep reinforcement learning in other robot tasks, such as motion planning and tactical decision-making for soccer robots. Additionally, we will further investigate how to apply these methods to real-world environments and address challenges and issues encountered in real scenarios. We hope that through continued research and exploration, we can make further contributions to the development and application of intelligent agent control. Future work can focus on advancing model capabilities, optimizing for practical use, and expanding the approach to new application areas. Explore application of end-to-end deep learning in more basketball skills like passing, dribbling, shooting under pressure. Integrate more sensing modalities like motion capture to enable more fluid autonomous dribbling and shooting motions.

In conclusion, this research successfully improved the shooting skills of a basketball robot using an end-to-end architecture and multi-modal perception with deep reinforcement learning. Our work provides new insights and methods for the application and development of intelligent agents in complex environments, demonstrating significant research significance and practical value. We are confident that through ongoing efforts and exploration, intelligent agents will achieve more remarkable progress and breakthroughs in the near future.

## Data availability statement

The original contributions presented in the study are included in the article/supplementary material, further inquiries can be directed to the corresponding author.

## Author contributions

JZ: Conceptualization, Formal analysis, Methodology, Resources, Writing—original draft. DT: Data curation, Funding acquisition, Investigation, Project administration, Writing—review and editing.
